# A novel homozygous R764H mutation in crumbs homolog 1 causes autosomal recessive retinitis pigmentosa

**Published:** 2013-04-05

**Authors:** Leila Tiab, Leila Largueche, Ibtissem Chouchane, Kaouthar Derouiche, Francis L. Munier, Leila El Matri, Daniel F. Schorderet

**Affiliations:** 1 Institute for Research in Ophthalmology (IRO), Sion, Switzerland; 2 Oculogenetic unit (UR 17/04), Hedi Rais Institute of Ophthalmology, Tunis, Tunisia; 3 Faculty of medicine, University Tunis El Manar, Tunisia; 4 Ophthalmology Department, Jules-Gonin Eye Hospital, University of Lausanne, Lausanne, Switzerland; 5 Faculty of Life Sciences, Ecole Polytechnique Fédérale de Lausanne, Lausanne, Switzerland

## Abstract

**Purpose:**

Retinitis pigmentosa (RP; MIM 268000) is a hereditary disease characterized by poor night vision and progressive loss of photoreceptors, eventually leading to blindness. This degenerative process primarily affects peripheral vision due to the loss of rods. Autosomal recessive RP (arRP) is clinically and genetically heterogeneous. It has been associated with mutations in different genes, including *CRB1* (crumbs homolog 1)*.* The aim of this study was to determine the causative gene in a Tunisian patient with arRP born to non-consanguineous parents.

**Methods:**

Four accessible family members were included. They underwent full ophthalmic examination with best-corrected Snellen visual acuity, fundus photography and fluorescein angiography. Haplotype analysis was used to evaluate homozygosity in the family to 20 arRP loci. All exons and intron-exon junctions of candidate genes not excluded by haplotype analysis were PCR amplified and directly sequenced.

**Results:**

The proband was a 43-year-old female patient. Best-corrected visual acuity was 20/63 (right eye) and 20/80 (left eye). Visual loss began during the third decade. Funduscopic examination and fluorescein angiography revealed typical advanced RP changes with bone spicule-like pigment deposits in the posterior pole and the midperiphery along with retinal atrophy, narrowing of the vessels, and waxy optic discs. Haplotype analysis revealed homozygosity with microsatellite markers D1S412 and D1S413 on chromosome 1q31.3. These markers flanked *CRB1*. Our results excluded linkage of all the other arRP loci/genes tested. Sequencing of the 12 coding exons and splice sites of *CRB1* disclosed a homozygous missense mutation in exon 7 at nucleotide c. 2291G>A, resulting in an arginine to histidine substitution (p.R764H).

**Conclusions:**

R764H is a novel mutation associated with *CRB1*-related arRP. Previously, an R764C mutation was reported. Extending the mutation spectrum of *CRB1* with additional families is important for genotype-phenotype correlations and characterization of the scope of mutation.

## Introduction

The term pigmentary retinopathy covers a set of hereditary diseases. Some are present at birth while others appear much later, around 40 or 50 years of age and even later. The primary symptoms are often difficulties with night vision. The retina gradually develops pigment spots, thus the name of the disease. This degenerative process initially affects peripheral rather than central vision, due to the loss of rods. Retinitis pigmentosa (RP; OMIM 268000) has a prevalence of about 1:4,000 [1]. RP is clinically and genetically heterogeneous with autosomal dominant (30%–40% of cases), autosomal recessive (50%–60% of cases), and X-linked inheritance (5%–15% of cases) [1,2]. A total of at least 36 genes and loci have been found to cause autosomal recessive RP (arRP). In three of the 36 previously reported arRP loci (RP22, RP29, RP32) the causative gene has not been discovered. Identification of a new gene or mutation allows the confirmation of the diagnosis and a better understanding of the familial pathology and the functional aspects implicated in the disease. Identifying new genes or mutations is also important for accurate genetic counseling and identifying potentially treatable forms.

Mutations in crumbs homolog 1 (*CRB1*) have been shown to result in retinitis pigmentosa and Leber congenital amaurosis (LCA), a severe form of retinal dystrophy, present at birth [3,4]. *CRB1* is a vertebrate homolog of the *Drosophila* crumbs. This gene consists of 12 exons and encodes crumbs, a protein of 1376 amino acids involved in the polarity of epithelial cells [5]. CRB1 is present in all retinal epithelial cells, rod and cone epithelial cells, and Müller glial cells in the adult mouse retina [6].

**Figure 1:**
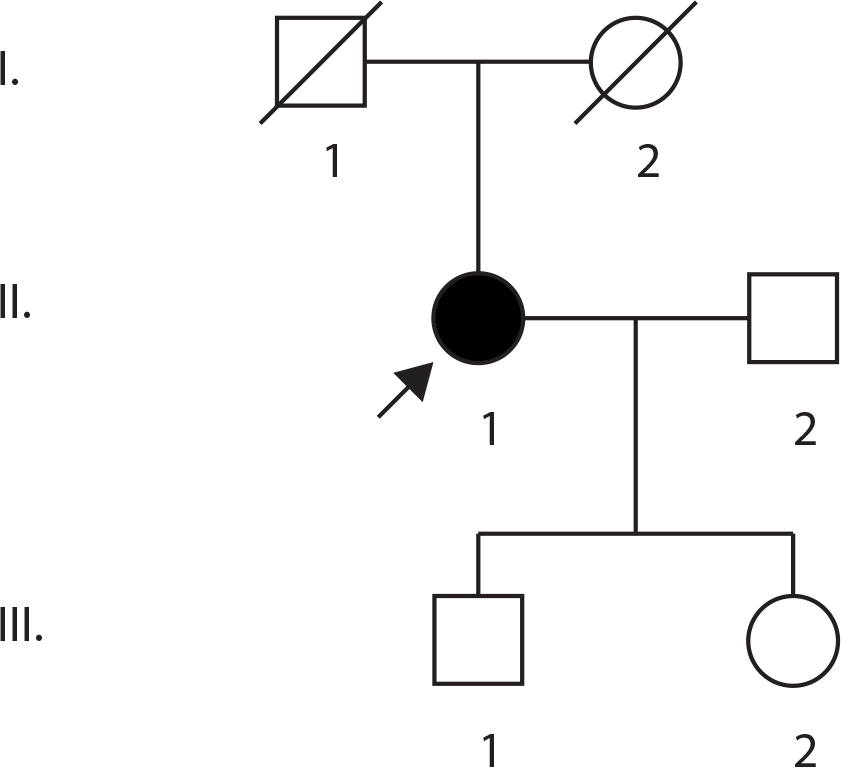
Pedigree structure of the investigated family. Squares and circles represent men and women, respectively. The arrow points to the proband (II:1) affected with retinitis pigmentosa.

In this study, we characterized a Tunisian family (Figure 1) with RP with an unknown, but probable, autosomal recessive mode of inheritance. To determine if the family was linked to one of the known arRP genes, two microsatellite markers were selected from the known genes and loci of arRP and used to genotype the members of this family. After linkage analysis, we excluded all arRP genes but the *CRB1* locus. Mutation screening of the 12 exons and splice sites of *CRB1* gene revealed a novel homozygous missense mutation.

## Methods

To assess the molecular origins of RP/LCA in Tunisia, 156 patients belonging to 67 families were enrolled. Among this cohort, 87 (36 females and 51 males) were affected and 69 (43 females and 26 males) were healthy. As recruitment of patients was not based on appearance of symptoms, the age of affected and non -affected individuals varied from less than 2 years to over 70 years. Accessible members were clinically assessed and underwent complete ophthalmic examination, including best-corrected visual acuity (BCVA) with Snellen chart and illiterate E chart, slit-lamp biomicroscopy, and fundus photography. In the proband, fluorescein angiography was performed (fundus camera: FA IMAGEnet, Topcon Corporation, Tokyo, Japan). Blood samples were obtained from participating family members after informed consent was received in compliance with the Declaration of Helsinki for research involving human subjects. The study was approved by the Hedi Rais Institute of Ophthalmology.

### DNA extraction

Total human genomic DNA was isolated with the DNA isolation Kit for Mammalian Blood (Nucleon Bacc2 genomic DNA Extraction, Amersham RPN 8502, GE Healthcare, Glattbrugg Switzerland).

### Haplotype analysis

Linkage and haplotype analyses were used to test linkage of the family to 19 arRP loci, including *CRB1, ABCA4, LRAT, USH2A, RP29, CERKL, CNGA1, CNGB1, EYS, FAM161A, SAG, MERTK, NR2E3, PDE6A, PDE6B, RGR, RHO, RLBP1*, *and TULP1.* Microsatellite markers flanking these loci were selected from (Appendix 1), amplified with PCR, and genotyped using an ABI 3100XL Genetic Analyzer (Applied Biosystems, Foster City, CA; with the Gene Mapper 2.5 software program from Applied Biosystems). Haplotypes were constructed by hand and checked for homozygosity.

### Mutation screening

Primer3 (v. 0.4.0) software was used to designed oligonucleotides primers from the DNA sequence of *CRB1*. These primers covered all the exons and splice junctions of *CRB1*, including the promoter (Table 1). Twelve exons and splice sites were analyzed in the proband after PCR amplification with the following conditions: initial denaturation for 5 min at 95 °C, followed by 35 cycles of denaturation at 94 °C for 1 min, annealing at 60 °C for 1 min, extension at 72 °C for 1 min, and final extension at 72 °C for 10 min. Amplicons were directly sequenced on an ABI 3100XL DNA sequencer, using the Big Dye Terminator Labeling Kit version 1 (Life Technologies, Zug, Switzerland). The frequency of the mutation was checked in 96 ethnically matched controls and in 96 Caucasian controls.

**Table 1:** Primers for amplification of exons of *CRB1*

Exons	Forward primer (5′ - 3′)	Reverse primer (5′ - 3′)	Annealing temp. (°C)
1	GTAGGGTGGGACAGAGATGG	AACTGACTGTTCACATTGACTGG	60
2	TTTTTCATTAGGATGAACCCAAC	CCATGTTGGCAGGAGTTCTT	60
3	CTCTGCCTTGTGCAACTTCC	TAAGCCGAGAACGTGAGAGC	60
4	CCATGGGTCTTGGGTTGATA	GCACCACAGCAGCAGAGTT	60
5	GATTCCCCTTACCAGCTCCT	GCACCACAGCAGCAGAGTT	60
6a	AACCTGAGCTATTCATGCACTTC	TGGTGTTGTGGGAAATGAAC	62
6b	GCAACAGGGATGTGTTTGTG	TTTCATAGCAGGCAGAAGCA	60
7a	TTCGTCTTCCATCCCTTCTG	GTCAGGTAGGCCACCAATGT	60
7b	GGAAATGTCCACTTGATATCTTTG	CTGGTGGGTCAGTAACATCATC	60
8	GCCCTTTTAGAAAGGAGTTGG	AGGCAAGAGGCCAGTCAGTA	60
9a	CATGTATCAAATAGTCAATATGCAATG	GTCTGGGACAGTGGGTCTGT	60
9b	ATTGCAAAGTGGCAACAGC	CAAGGGACAGGAGCAATGAT	60
9c	CAGTGGTCACTGGCTGTTTG	CAGTGTCACCCTGTTCAGCA	60
10	CCTCCAGCAGGAGCTTTTTA	GCATAGATTTTCCTATGGGAACTG	60
11	AGACTGTGCTGTTCCAGAGAGA	TTTTTCATTTGGGAATGTTCAA	60
12	GCTTGCTCTGGTTGGTCTTC	TTTTAAGGTGGTCAAAGGAATCA	60

## Results

### Clinical findings

**Figure 2:**
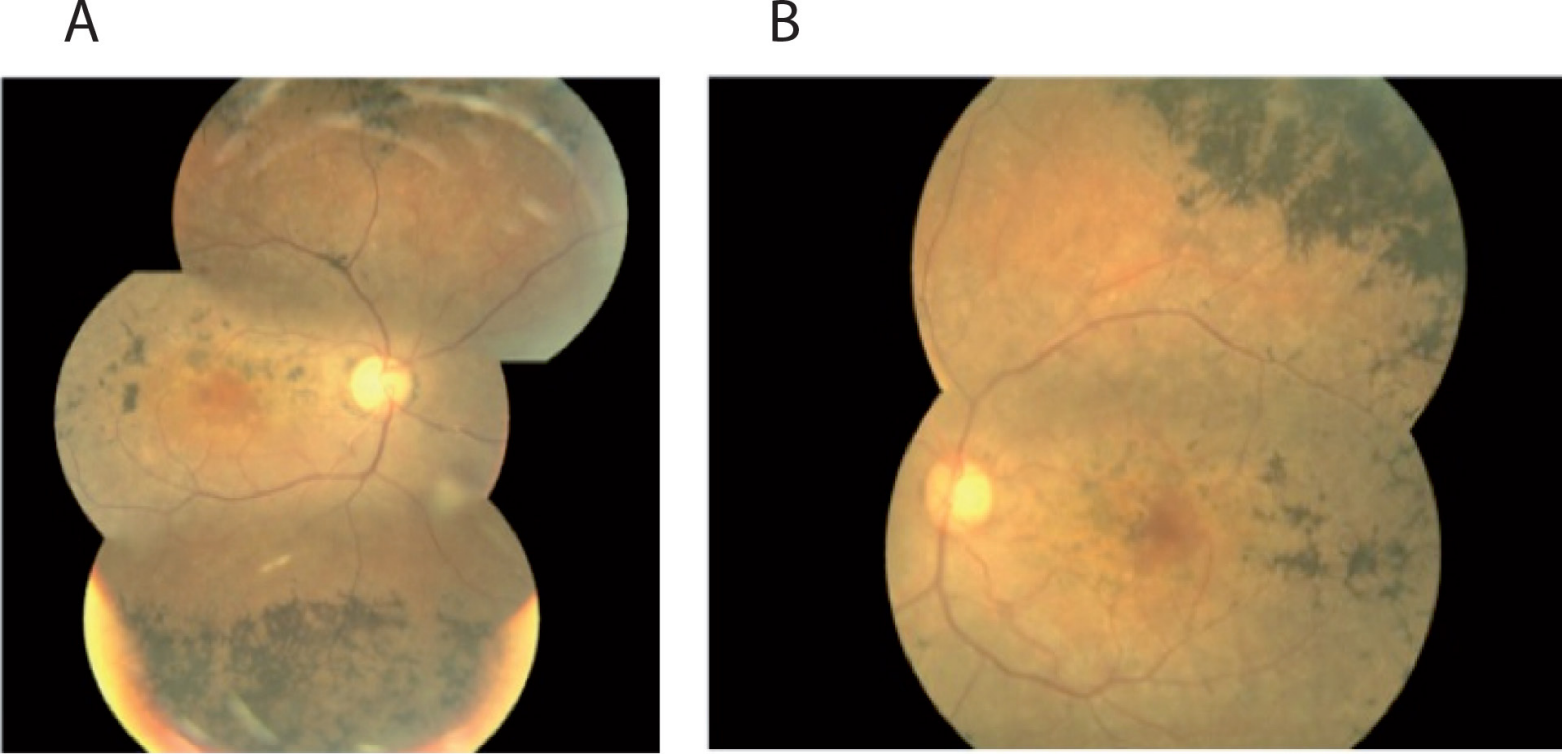
Color fundus photographs of the proband at 43 years of age. Left (**A**) and right (**B**) eyes showing typical advanced retinitis pigmentosa changes with bone spicule-like pigment deposits in the posterior pole and midperiphery along with retinal atrophy, narrowing of the vessels, and waxy optic discs.

The proband (II:1) was born to unrelated parents, and their marriage was not consanguineous (Figure 1). The primary symptom of vision problems, reduced visual acuity, occurred at the age of 37 years, and she was 40 when she came for her first ophthalmic visit at the Institute. At that time, BCVA was 20/63 in the right eye and 20/80 in the left. The anterior segment was normal in both eyes. There was no cataract or refractive error. Funduscopic examination and fluorescein angiography revealed typical advanced retinal changes with bone spicule-like pigment deposits in the posterior pole and the midperiphery along with atrophy, narrowing of the vessels, and waxy optic discs (Figure 2). No additional symptoms or ocular defects such as strabismus or nystagmus were observed in the proband. Her husband and both children were clinically examined. No abnormalities were found in all three individuals. Visual acuities were 10/10, and fundus photography was normal except in the boy, who presented hypertrophy of the pigment epithelium in the temporal superior quadrant of the left eye.

### Molecular analysis

**Figure 3:**
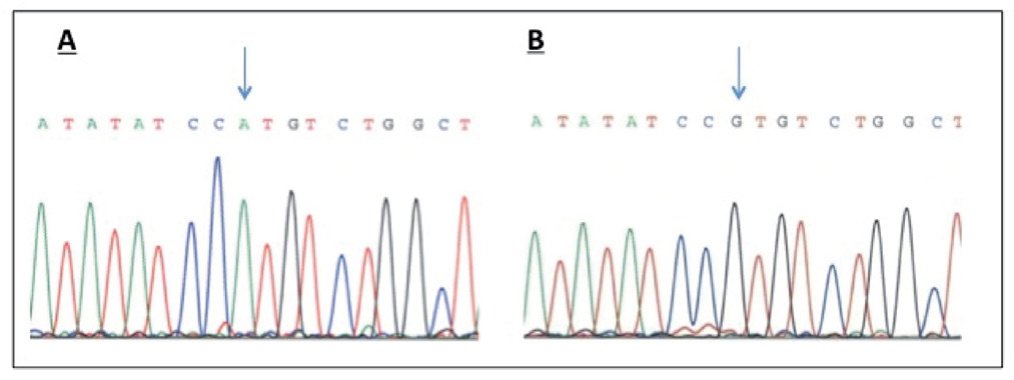
Partial electropherogram of exon 7 of *CRB1*. **A**: Electropherogram of the proband showing a c. 2291 homozygous G to A transition mutation in exon 7 of the crumbs homolog 1 gene leading to a p.Arg764His amino acid change. **B**: Normal sequence. Arrows indicate the position of the mutated nucleotide.

**Figure 4:**
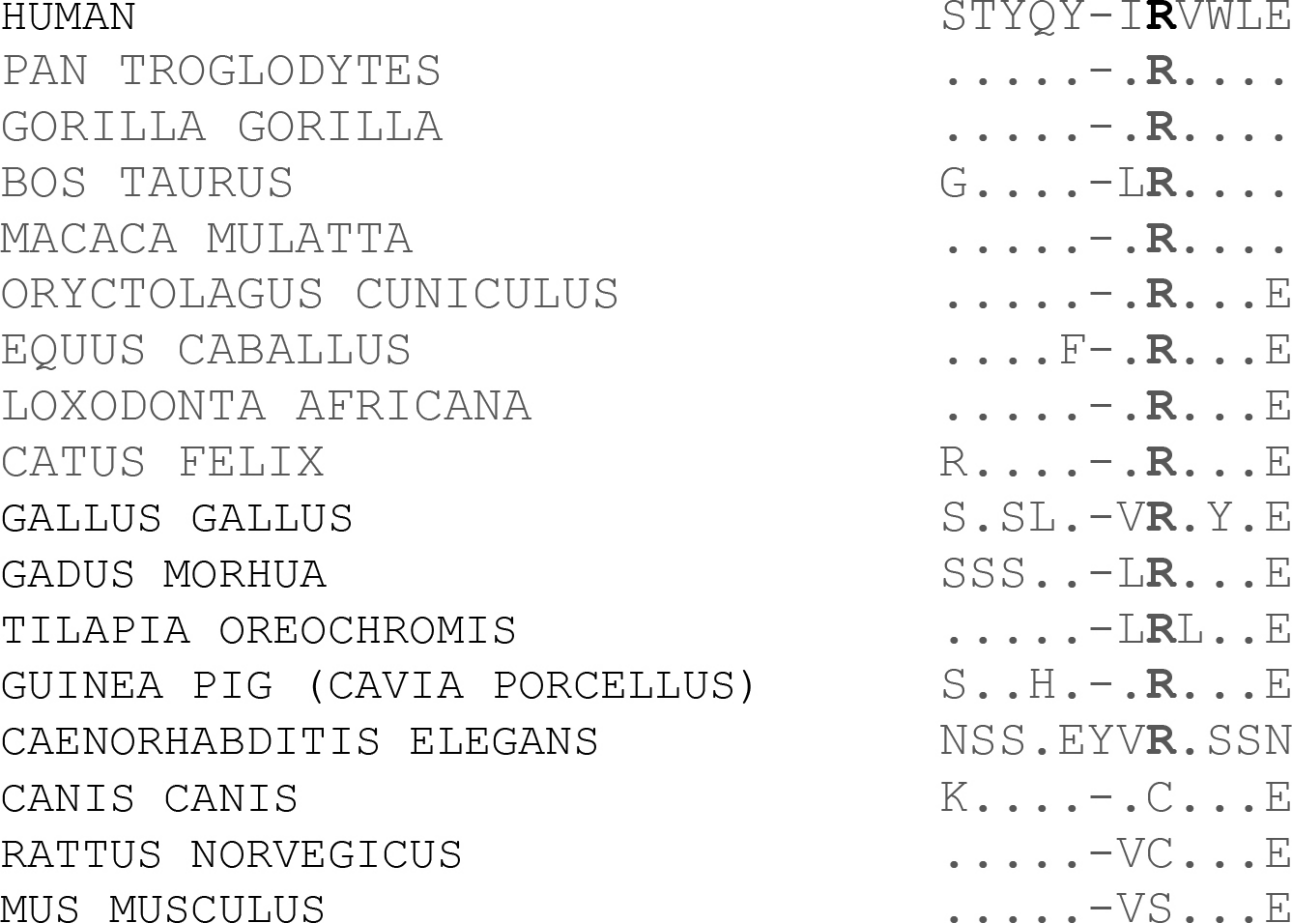
Alignment of various *CRB1* orthologs. Human R764 is in bold. Conserved amino acid are replaced by a dot.

Mutation analysis for autosomal recessive RP did not reveal any previously reported mutation. Following these results, we tested the most frequent arRP loci and genes by linkage in 14 families compatible with LCA out of a cohort of 67 families. Haplotype analysis revealed homozygosity in five families with microsatellite markers D1S412 and D1S413 on chromosome 1, two markers flanking *CRB1*. Our analysis excluded linkage of the other arRP loci/genes. Sequencing all the exons and intron-adjacent regions of *CRB1* in the probands of these families did not identify any mutation, except the one family reported here. In that family, sequencing *CRB1* disclosed a missense homozygous mutation in exon 7 at nucleotide c. 2291G>A, resulting in the substitution of histidine (His) for arginine (Arg) at position 764 (p.Arg764His; Figure 3). Examination of the pathogenicity of the Arg764His mutation with PolyPhen generated a score of 0.975 (probably damaging), close to the maximum value of 1.00, while Sorting Intolerant From Tolerant (SIFT) predicted a tolerated change with a score of 0.15. This mutation was not observed in 96 ethnically matched controls or in 96 Caucasian individuals. Alignment of *CRB1* showed that R764 was conserved among various species, from apes to *Caenorhabditis elegans*. However, in several mammals, including the dog, rat, and mouse, R764 was not conserved. In these three species, the basic amino acid was changed to a hydrophobic (cysteine) or polar (serine) amino acid (Figure 4).

## Discussion

**Figure 5:**
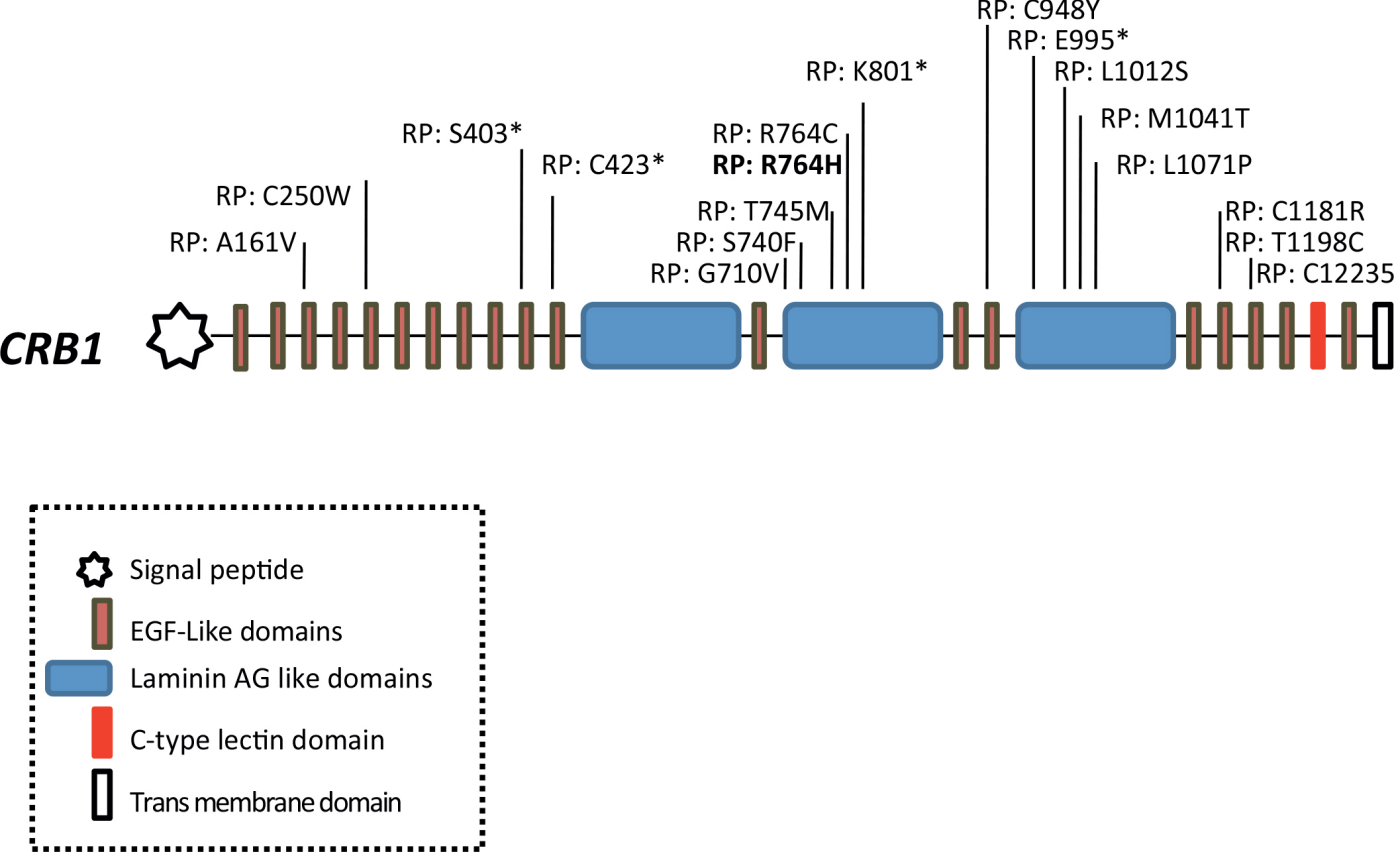
Schematic representation of the crumbs homolog 1 gene with retinitis pigmentosa–causing mutations. The reported mutation is in bold.

Mutations in *CRB1* have been reported in patients with LCA [7,8], in two forms of retinitis pigmentosa, with or without preservation of the para-arteriolar retinal pigment epithelium [9] and in RP with Coats-like exudative vasculopathy [10,11]. *CRB1* is expressed in the human retina, brain, and fetal brain, and CRB1 is an extracellular protein with a signal peptide, 19 epidermal growth factor–like domains and three laminin A G-like domains and C-type lectin (CTL) domain [12] (Figure 5). These domains interact with other extracellular or transmembrane proteins [13]. CRB1 regulates the polarity of epithelial cells by participating in assembling the zonula adherens region. In *Drosophila*, defects in crb cause an abnormal shape of the photoreceptors, and in the mouse, a frameshift mutation at nucleotide 3481 causes the rd18 phenotype. To date, more than 70 missense/nonsense mutations have been identified in *CRB1* in patients with retinal dystrophies, and 19 variants have been reported associated with RP12 (HGMD) [14]. All these mutations map on the extracellular domain precisely on the epidermal growth factor– and laminin A G-like domains [3]. These domains are conserved from flies to mammals [13]. The Arg764His mutation is localized on the second laminin A G-like domain (Figure 5). This domain is an 180-amino-acid-long domain found in a large and diverse set of extracellular proteins [15,16]. Laminin AG-containing proteins appear to have a wide variety of roles in cell adhesion, signaling, migration, assembly, and differentiation [17]. The CRB1 protein is located at the level of the photoreceptor inner segment more precisely at the adherens junctions that connect rod and cones photoreceptor cells with each other and with Müller glial cells [18]. As CRB1 is inserted through the cell membrane, this protein probably serves important functions inside and outside the photoreceptor cell. The 37-amino-acid cytoplasmic segment is known to act as an anchor for several proteins, such as MPP5 or PALS1 [19].

p.Arg764His is a novel *CRB1* mutation and represents the first homozygous mutation described in a patient originating from North Africa associated with arRP. This mutation also represents the second missense mutation at residue R764 [3,20]. The p.Arg764His mutation is most likely pathogenic as mutations in codon 764 generating cysteine mutants have already been reported. In addition, the p.Arg764His mutation was not found in 96 ethnically matched or in 96 European control individuals, and strong pathogenicity was predicted by PolyPhen. Interestingly, SIFT predicted a tolerated change. This may be because R764 was not conserved in the rat, dog, and mouse. In these first two species, arginine is replaced by cysteine, the same change observed by den Hollander et al. and Henderson et al. in five patients [3,11,20]. All patients were compound heterozygotes: three with an S403X and one with an E995X mutation. The second mutation was not found in the last patient. In addition to RP, three of these patients had Coats-like exudative vasculopathy. Genotype-phenotype correlations are difficult to establish for CRB1 mutants. However, three out of four patients with a first missense mutation at codon R764 had a null second allele. This is in contrast with the patient we described. Lack of haploinsufficiency and the presence of two missense alleles may be responsible for the late-onset retinopathy observed in this patient.

Although we have not been able to document consanguinity between the parents of the proband, it is unlikely that these two alleles originated from independent mutations as the proband displayed a homozygous chromosomal region that was at least 4.6 Mb in length. Homozygosity mapping may therefore still be used in specific ethnic backgrounds despite a lack of overt consanguinity.

Precise identification of the molecular mutations in patients with arRP is important for genetic counseling and for future therapies. Loss-of-function mutations in slow-developing RP will most likely be among the first to benefit from gene therapy as already shown for *RPE65*. Patients such as the one described here might be good candidates for such treatment.

## Appendix 1: List of genes/loci and flanking markers.

To access the data, click or select the words “Appendix 1.”
